# Visceral Adiposity and Cancer: Role in Pathogenesis and Prognosis

**DOI:** 10.3390/nu13062101

**Published:** 2021-06-19

**Authors:** Lucilla Crudele, Elena Piccinin, Antonio Moschetta

**Affiliations:** 1 Department of Interdisciplinary Medicine, University of Bari “Aldo Moro”, 70124 Bari, Italy; lucilla.crudele@uniba.it (L.C.); elena.piccinin@uniba.it (E.P.); 2 Department of Biomedical Sciences and Human Oncology, University of Bari “Aldo Moro”, 70124 Bari, Italy; 3 Department of Basic Medical Sciences, Neurosciences and Sense Organs, University of Bari “Aldo Moro”, 70124 Bari, Italy; 4 INBB, National Institute for Biostructures and Biosystems, 00136 Rome, Italy; 5 National Cancer Center, IRCCS Istituto Tumori Giovanni Paolo II, 70124 Bari, Italy

**Keywords:** obesity, cancer, visceral adiposity, Mediterranean diet, fasting, lifestyle interventions

## Abstract

The prevalence of being overweight and obese has been expanded dramatically in recent years worldwide. Obesity usually occurs when the energetic introit overtakes energy expenditure from metabolic and physical activity, leading to fat accumulation mainly in the visceral depots. Excessive fat accumulation represents a risk factor for many chronic diseases, including cancer. Adiposity, chronic low-grade inflammation, and hyperinsulinemia are essential factors of obesity that also play a crucial role in tumor onset. In recent years, several strategies have been pointed toward boundary fat accumulation, thus limiting the burden of cancer attributable to obesity. While remodeling fat via adipocytes browning seems a tempting prospect, lifestyle interventions still represent the main pathway to prevent cancer and enhance the efficacy of treatments. Specifically, the Mediterranean Diet stands out as one of the best dietary approaches to curtail visceral adiposity and, therefore, cancer risk. In this Review, the close relationship between obesity and cancer has been investigated, highlighting the biological mechanisms at the basis of this link. Finally, strategies to remodel fat, including browning and lifestyle interventions, have been taken into consideration as a major perspective to limit excess body weight and tumor onset.

## 1. Obesity and Cancer

Overweight and obesity are defined by an abnormal and excessive fat accumulation that develop when caloric intake by meals exceeds energy expenditure from physical activity and cells metabolism. In clinical practice, obesity is commonly assessed by expressing body weight as a function of height, according to World Health Organization (WHO) classification of Body Max Index (BMI). BMI between 25 kg/m^2^ and 29.9 kg/m^2^ is categorized as overweight, whereas greater values define obesity condition [1]. According to data published by the WHO, 39% of adults were overweight and 13% were obese in 2016 [2]. Consequently, being obese is a well-known risk factor for cardiovascular and metabolic diseases, the burden of obesity in health systems is becoming more relevant every day. Moreover, metabolic dysregulations associated to obesity and, more specifically, to visceral adiposity has been found to play a crucial role in tumor biology, affecting cells proliferation and spreading, as well as response to therapy [3], and therefore are studied as cancer-related conditions in term of risk, prognosis and mortality [4].

### 1.1. Incidence: When Obesity Promotes Cancer

In 2012, about 3.6% of all new cancer cases in adults were associated with high BMI assessed ten years before cancer diagnosis. Corpus uteri, postmenopausal breast and colon cancers accounted for 63.6% of tumors linked to high BMI [5]. Renehan et al. [6] systematically reported that a 5 kg/m^2^ increase in BMI can be considered as a risk factor for several solid and non-solid cancer (cancer of thyroid, kidney and colon, esophageal adenocarcinoma, multiple myeloma, leukemia and non-Hodgkin lymphoma) in both sexes. In men, this increment in BMI has been associated with a greater risk also for rectal cancer and melanoma, whereas in women it raises the possibility of developing gallbladder, pancreatic, endometrium neoplasms, and breast cancers, especially in post-menopausal patients. Gender difference was nevertheless significant only in colorectal cancer. This finding is supported by a Mendelian randomization study [7] on the association between BMI and colorectal cancer incidence: risk was 22% higher among men per 4.2 kg/m^2^ increase in BMI and 9% higher among women per 5.2 kg/m^2^. In 2016, the International Agency for Research on Cancer (IARC) Working Group added hepatocellular carcinoma, meningioma, gastric cardia and ovarian cancers to the aforementioned obesity-associated tumors [8]. Obesity has a potential role in the etiology of gastric and esophageal cancers [9], and abdominal obesity has been associated with an increased risk of breast cancer [10] (Table 1). 

It is noteworthy that BMI-cancer association seems to be specific only for some histological types of tumors considering a specific site [11]. For instance, obesity is more strongly associated with type I endometrial cancer than type II [12], to papillary subtype of thyroid carcinoma [13], cardia gastric cancer [14], and esophagus adenocarcinoma [15] among other histological types.

However, several studies depicted and inverse obesity-cancer association for lung cancer, a phenomenon called “obesity paradox”. Importantly, the misinterpretation that obesity might be protective against cancer has to be avoided, since potential confounders such as smoking habit and its influence on body weight [16], in addition to possible methodological flaws [17], must be taken into consideration when we are discussing the obesity paradox.

Albeit increased BMI is the most common studied obesity-linked condition, other biomarkers have been detected as hallmarks of an increased susceptibility to cancer. BMI and fasting insulin have both been identified as risk factors for breast cancer and are associated with late-stage disease and poor prognosis [18]. Moreover, a higher serum level of C-peptide, a measure of insulin secretion usually elevated in insulin resistance status, has been indicated as a risk factor for colorectal cancer development [19].

Finally, the hormonal changes that also occur in obesity can be associated with cancer onset. In this view, particular emphasis has been put on estrogens and menopausal status since they are strictly related with higher incidence of female specific cancers. Post-menopausal status in obese women increases risk of developing breast cancer, whereas ovarian cancer incidence is greater in pre-menopausal ones [15]. In addition, menopausal hormone replacement therapy positively affects the association between BMI and post-menopausal breast and endometrial cancer [11] while it reduces colorectal cancer rates by 37% [20]. 

### 1.2. Obesity and Cancer: Prognosis and Mortality

Although many efforts have been made in the past, identifying prognostic factors in cancer is still an important goal of ongoing studies. BMI, body weight, adult weight gain, and fat distribution, separately or taken in combination, may be considered as risk factors for poor cancer prognosis [21]. Obesity affects the development of aggressive and nonaggressive cancer differently; for instance, BMI ≥30 kg/m^2^ has been associated with a 29% increased risk of high-grade cancer in prostate [22]. In 2010, 3.9% of cancer mortality was linked to high BMI measured ten years earlier [23], and 15% to 20% of deaths in cancer patients have been related to overweight and obesity conditions [24]. Albeit outside of breast cancer the relationship between BMI and cancer survival is not so consistent [25], probably because of remarkable variations among studies, emerging data suggest that obesity can also represent an increased risk of cancer recurrence [24]. 

**Figure 1:**
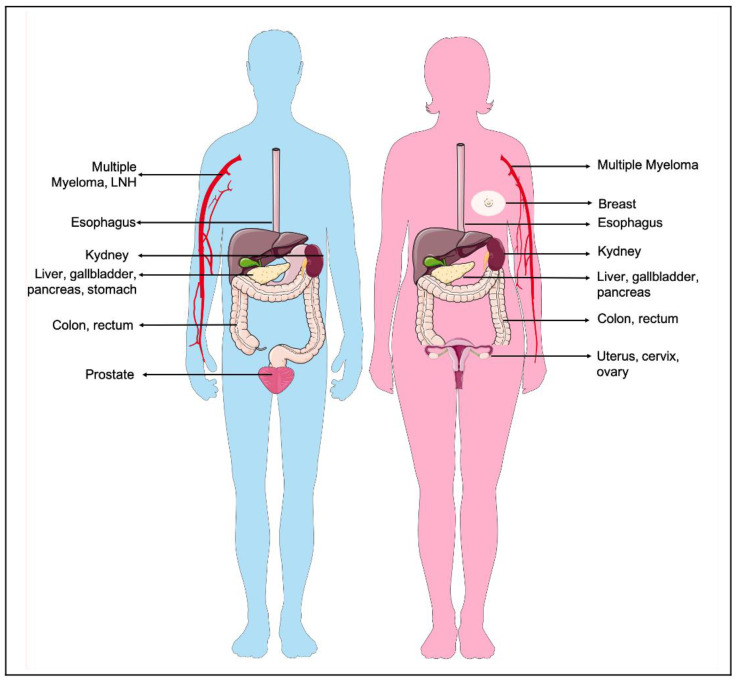
Overweight and obesity increased the risk for developing cancer in different sites. Body fat has been associated with increased risks for a number of cancers that occur in different sites according to sex. The cancer types depicted in the figure displayed increased mortality rate if in association with an obesity condition. Parts of the figure were drawn by using pictures from Servier Medical Art. Servier Medical Art by Servier is licensed under a Creative Commons Attribution 3.0 Unported License.

Calle et al. [26] studied the association between overweight and cancer-related deaths during 16 years of follow up, in a cohort of more than 900,000 U.S. adults who were free of cancer at the enrolment. The study revealed that the risk of dying of cancer was 50% to 62% higher in people with highest BMI. Obese male subjects had elevated mortality rates of liver cancer (the highest one), stomach cancer, non-Hodgkin’s lymphoma, and prostate cancer. On the contrary, in female subjects a positive association between weight and cancer death was detected for uterus, cervix, breast, liver, and ovarian cancers. Both high BMI men and women showed high mortality rates for cancers affecting the gastrointestinal system (pancreas, esophagus, gallbladder, colon, and rectum) as well as kidney cancer and multiple myeloma (Figure 1). By contrast, obesity displayed no significant associations with mortality due to brain cancer, bladder cancer, and melanoma in both sexes. In addition to this direct obesity-mortality association, a large amount of epidemiological evidence suggests a close association between obesity comorbidities, such as cardiovascular diseases and diabetes, and non-cancer related deaths in cancer survivors [27]. These observations highlight that the importance of weight management and healthy lifestyle not only prevent cancer, but also prolong the disease-free survival state.

Particularly in a large population study, diabetes was associated with increased hazard-ratio for cancer specific death [28]. Notably, insulin resistance in women affected by breast cancer displayed a two-fold increased risk of distance recurrence and a three-fold decreased survival [29], to the point that C-peptide has been proposed as prognostic factor in breast cancer [30].

Furthermore, the metabolic syndrome [31], a well-known obesity associated condition [32], increases the risk of developing [33] and having relapse in colorectal cancer [34,35].

Finally, obesity can interfere with therapies and contributes to morbidity from chemotherapy toxicities, thus promoting worst prognosis and mortality [27]. On the other hand, physicians usually under-dose cancer therapies when doses—calculated according to body surface area—seem to be too high [36], despite that no evidence has found for this practice of dose-capping [37]. Furthermore, obese patients undergoing oncologic surgery usually required longer operative and anesthetic times [36] and, on account of technical challenges, are offered open surgery more frequently than laparoscopic, although the latter has a better overall survival rate [38]. Finally, obesity represents a risk factor for poor wound healing, postoperative infections, and long-term surgical complications, like lymphedema [27].

## 2. Obesity and Cancer: Deepening the Relationship

**Figure 2:**
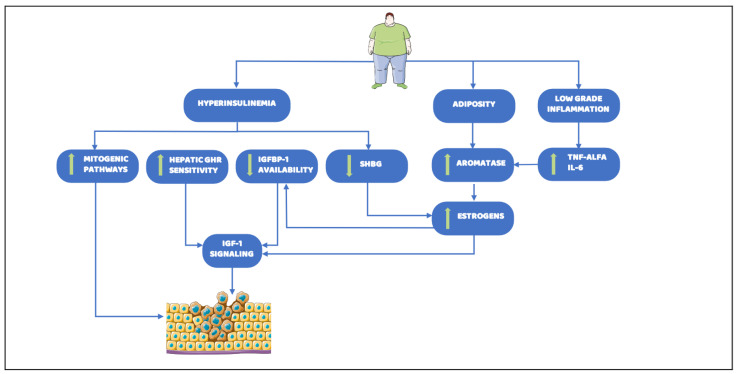
Main biological mechanisms linking obesity and cancer risk. Obesity constitutes major determinants of the increasing incidence and prevalence of cancer. Several aspects underlying obesity, such as hyperinsulinemia, adiposity, and low grade inflammation, have been found as the major causes leading to cancer onset. Downward arrow indicates a decrease, whereas upward arrow indicates an increase. Abbreviations: GHR: Growth Hormone Receptor; IGFBP-1: Insulin-like growth factor-binding protein 1; SHBG: Sex Hormone Binding Globulin; TNF-alfa, Tumor Necrosis Factor-alfa; IL-6: Interleukin-6; IGF-1: Insulin Growth Factor-1. Parts of the figure were drawn by using pictures from Servier Medical Art. Servier Medical Art by Servier is licensed under a Creative Commons Attribution 3.0 Unported License.

The complexity of epidemiological evidence about the relationship between obesity and cancer reflects the wide interplay among metabolic pathways involved in cancer development, progression, and pharmacological responsiveness. To date, a plethora of circulating factors and molecular alterations, whereby obesity can lead to carcinogenesis, have been detected. Despite this, primarily relevant mechanisms are not well understood. Here, we provide an overview of the principal identified factors that link altered metabolism to tumor onset and progression, focusing on three typical and deeply interplaying cornerstones of obesity which are also possible early diagnostic biomarkers and strong predictors of cancer risk: adiposity, chronic low-grade inflammation, and hyperinsulinemia (Figure 2).

### 2.1. Adiposity

In humans, two main kinds of adipose tissue (AT) exist: white (WAT) and brown (BAT). Recently, the existence of a third kind has been proposed, i.e., “brite” or “beige” AT, described later in this manuscript [39]. BAT is mainly located in interscapular, perirenal, and deep neck regions of newborns [40]. In human adults, BAT is only present in particular conditions, such as the exposition to low temperature. Indeed, the main function of BAT is the thermogenesis—the dissipation of energy to produce heat. Differently from BAT, WAT is located in subcutaneous and visceral depots, and is present all lifelong. The primary role of WAT is storing the excessive amount of energy disposal as fat, mainly derived from serum triglycerides (TAG) and *de novo* lipogenesis (DNL) from exceeding carbohydrates or other non-fat precursors. Indeed, a low-fat, high-carbohydrate diet increases DNL in liver [41] and in WAT [42]. Notably, DNL has been found to be exacerbated in several cancer tissues and correlates with poorer prognosis [43]. Fatty acid synthase (FASN), the key enzyme of DNL process, is overexpressed and hyperactivated in cancers with a high risk of disease recurrence and death [44]. This observation shed a light on cancer metabolism, revealing that exacerbated lipogenesis and FFAs metabolism are crucial for cancer onset and progression, along with the well-established glycolytic and glutaminergic pathways [45,46].

By functioning as a fat depot, WAT prevents lipotoxicity, a detrimental process determined by excess of FFAs that can alter cell membrane structure and functions, creating an inflammatory environment, which finally leads to cell dysfunction and death. In obesity state, lipotoxicity has been found to contribute to increase macrophage infiltration into AT, insulin resistance, and hepatic steatosis [47].

During prolonged calorie abundance, adipocytes hyperplasia (recruitment of preadipocytes and differentiation to mature cells) and hypertropia (enlargement of existing cells) occur in healthy subjects to store the increase amount of fat. The differentiation process from mesenchymal stem cells to mature adipocytes is called adipogenesis and is elegantly regulated by transcription factors, among which peroxisome proliferator-activated receptor γ (PPARγ) is the most critical for the process. Notably, PPARγ is the target of new and highly efficacious class of insulin-sensitizing drugs known as thiazolidinediones [48]. The adipocytes commitment and recruitment in subcutaneous adipose tissue (SAT), the largest WAT depot, avoid fat accumulation in ectopic tissues (i.e., liver where fat leads to non-alcoholic fatty liver disease (NAFLD), heart and muscles), as well as in visceral adipose tissue (VAT), which is mainly distributed in omental (hangs off the stomach), mesenteric (associated with the intestine), retroperitoneal, gonadal, mammary, and pericardial depots. When SAT maximum expansibility (maximum hypertrophy of pre-existing adipocytes and failure in adipogenesis) is achieved, calories surplus, due to excess energy intake and/or reduced energy expenditure, accumulates as fat in VAT, leading to its expansion. A marker of this ectopic fat accumulation in humans is the increased abdominal obesity [4]. Men are more prone to visceral adiposity with respect to women, who tend to accumulate fat in the lower part of the body [49]. The different distribution of fat between the two sexes may explain the diverse metabolic risk, higher in men, [50] and gender incidence in some types of cancer [51]. Differently from the SAT, VAT is closer to internal organs and directly connected to portal vein [52]; it is more metabolically active [53] and produces more adipokines [54]. Importantly, behind its well-known associations to glucose intolerance, dyslipidemia, and hypertension [55], VAT disproportionate expansion is deleterious because its amount positively correlates with tumorigenesis. For instance, omental adipocytes displayed significant more efficiency in promoting invasion of ovarian cancer cells than subcutaneous ones [56]. VAT accumulation and central (not general) obesity are specifically associated to cancer risk and prognosis through hyperinsulinemia [57,58], whose pathogenesis could reside in adipogenesis dysregulation which occurs when excessive AT develops [59]. Indeed, the dysfunction and enlargement of adipocytes, the decrease expression of lipogenic genes, and the expansion of VAT are all potential mechanisms underlying the obesity-related insulin resistance [60]. Additionally, the excess of adiposity and the dysregulation in adipogenesis lead to adipocytes hypoperfusion and hypoxia, which explain the impaired production of adipocytokines and metabolic syndrome development in obesity [41]. Moreover, hypoxia increases adipocytes death, leading to a persistent state of damage that enhances macrophages infiltration and causes low grade chronic inflammation [61], which is an hallmark of obesity [62]. Nevertheless, WAT is more than a fat depot, since it provides paracrine signals, secretes adipokines as a large endocrine organ, and is involved in insulin sensitivity and thermogenesis.

### 2.2. Adipokines and Chronic Low-Grade Inflammation

Chronic inflammation is a cornerstone in both obesity and tumorigenesis [63,64], thereby dysfunction in adipokines production represents a possible biological explanation for cancer promotion in obesity [65]. Adipokines [e.g., adiponectin and leptin, Interleukin-6 (IL-6) and Tumor Necrosis Factor-α (TNF-α)] are biologically active polypeptides secreted by WAT. Upon being released in the bloodstream by visceral fat, adiponectin promotes fatty acid oxidation and protects against insulin resistance [66], since it decreases glucose and insulin levels by increasing tissues insulin sensitivity and glucose uptake in feeding conditions. Also, adiponectin plays a pivotal role in regulation of endothelial function [67] and tumor growth [68]. Furthermore, adiponectin is important in preventing inflammation [69], as demonstrated by the increased association with pro-inflammatory effects due to enhance IL-6 and TNF-α production in hypo-adiponectinemia [70]. Notably, adiponectin serum level inversely correlates with BMI, probably because insulin resistance status, associated to AT inflammation [71], reduces its release. According to recent meta-analysis, adiponectin levels are inversely associated to breast [72] and colorectal [73] cancers. Contrarily to adiponectin, circulating level of leptin displays linear positive correlation with BMI, fat cell volume, and plasma insulin [74], which positively regulates leptin genes expression [57]. Besides its well-known action as anorexigenic hormone, leptin stimulates pro-inflammatory cytokines production [75], promotes angiogenesis [76] and acts as a growth factor in tumorigenesis, thus contributing to development of more aggressive cancers [77]. Moreover, leptin plays a key role in breast cancer development and treatment response, particularly in obese women [78]. However, leptin does not affect metastatic spread so its prognostic effect in cancer is not so consistent [79]. Also IL-6, which positively correlates with body mass and FFAs concentration [80], has been proposed as a prognostic factor in breast cancer, since it promotes cell migration and the increase of aromatase activity [81]. Overproduction of IL-6 and TNF-α in obesity is mainly due to the augmented infiltration of macrophages [i.e., Adipose tissue macrophages (ATM)] within WAT [82]. Both M1 and M2 infiltrating macrophages are present in fat: M2 macrophages display anti-inflammatory properties, whereas M1 macrophages enhance inflammation. Diet-induced obesity leads to a shift from a M2-polarized state to a M1 proinflammatory state, thus highlighting once more the close association between accumulation of fat and inflammation [83]. Importantly, increased IL-6 levels have been found also in subjects fed with high-sugar, high-fat and pro-inflammatory diet [84], opening new perspectives about dietary ability in modulating inflammation. Given that IL-6 and soluble tumor necrosis factor receptor 2 (sTNF-R2) together may be useful markers to predict cancer development [85], these findings corroborate the evidence that chronic low-grade inflammation related to central obesity increases risk of malignancy.

Beside this, it is fundamental to underscore that macrophages implicated in tumorigenesis are triggered by chronic inflammation in AT and act by different pathways from tumor-associated macrophages (TAMs), which instead are responsible for progression and metastasis via their interplay with tumoral microenvironment [86]. The paracrine and autocrine mechanism, whereby chemokines sustain the growth and development of the tumor, has been shown in prostatic cancer [87] and remarks the microenvironment involvement in tumorigenesis [88]. In the contest of this pro-tumor environment, adipocytes possess a pivotal role. For instance, mammary AT influences breast cancer promotion and invasion because fat tissue surrounding cancer possesses high levels of aromatase enzyme activity [89].

### 2.3. Hyperinsulinemia

In healthy conditions, insulin promotes fat storing in WAT by enhancing adipogenesis, stimulating glucose cells uptake and lipogenesis, while inhibiting lipolysis. Insulin also increases the uptake of fatty acids derived from circulating lipoproteins [90]. Obesity causes insulin resistance and chronic hyperinsulinemia, which seem to be critical in the association between cancer and adiposity [91]. Chronic hyperinsulinemia might lead to neoplasm development directly acting as a mitogenic factor itself [92], but also through its deep interplay with sex hormones and other growth factors pathways, e.g., insulin growth factor 1 (IGF-1) [93]. IGF-1 is a master regulator of cell proliferation, differentiation, and apoptosis [94], being pro-mitotic and anti-apoptotic [95]. IGF-1 bioavailability depends on IGF-binding protein-1 (IGFBP-1), to which it is bound to circulate in blood. Consequently, alterations in IGF-1 and IGFBP-1 levels affect the balance between cell proliferation and apoptosis, finally leading to tumor development. The majority of the circulating IGF-1 production take place in the liver, and it is regulated by the Growth Hormone (GH). Of note, GH is also likely to promote adipogenesis [96]. Although GH levels are reduced in obesity, hyperinsulinemia raises density and sensitivity of hepatic GH-receptors in diabetic and obese patients [97], leading to overproduction of IGF-1 [98] and increasing its availability by decreasing IGFBP-1 levels [99]. Thus, tumor growth is enhanced in response to increased signaling by insulin and IGF-1, conferring selective advantage to cancer cells, especially in hyperinsulinemia associated conditions. Insulin receptors are overexpressed in malignant epithelial cells of breast carcinoma [92] and IGF-1 receptors are overexpressed in many tumor cell types [19,95]. Notably, IGF-1 circulating level are positively associated with an increased risk of colorectal and prostate cancers, as well as with premenopausal breast cancer, although the strength of these associations varies by cancer site [100,101,102]. However, it is important to note that in some tissues IGF-1 synthesis is regulated by other hormones apart from GH [95]. Particularly, the estrogens are involved in IGF-1 signaling by increasing number and affinity of IGF1-receptors and decreasing IGFBP-1 production. Consequently, similarly to insulin, the estrogens promote cell proliferation and inhibit apoptosis. The ovaries are the major source of estrogens in premenopausal women. Instead, in post menopause subjects, estrogens mainly derive from the peripheral conversion of adrenal androgens via adipose aromatase, whose levels depend on fat amount as well as on TNF-α and IL-6 stimulation [103]. Notably, the peripheral conversion of androstenedione to oestrone observed in obese subjects is not the only responsible for the increase estrogens bioavailability. Indeed, insulin also downregulates sex-hormone-binding globulin (SHBG) hepatic production, thus increasing the estrogen activity [98]. Therefore, obese subjects and post-menopausal women are characterized by higher levels of circulating estrogens than normal individuals. In men, obesity-associated low levels of testosterone and SHBG, coupled with increased estrogens, have been proposed to be with high-grade prostate cancer [22]. Similarly, since in post-menopause ovarian sex hormones synthesis is suppressed, increased peripheral estrogens production in obese women is the main proposed explanation for higher breast cancer incidence [104]. As a consequence of high estrogen levels, aromatase inhibitors in chemotherapy may be less effective when administrated at normal doses in obese patients, thus affecting the prognosis of breast cancer [105]. Contrarily, progesterone opposes estrogen-related actions, reducing inflammation, enhancing apoptosis and cell differentiation, and increasing IGFBP-1 production. Differently from estrogens, progesterone levels are reduced in obese individuals. Therefore, an “unopposed estrogen” action may explain why cancer risk is increased also in pre-menopause obese women, as it occurred for endometrial cancer [106].

## 3. Perspectives into Visceral Adiposity and Cancer

Since obesity affects cancer incidence and prognosis, accurately identifying subjects with higher risk via both anthropometric and metabolic assessment is overriding. In this view, it could be intriguing to study if central obesity *per se* is sufficient to increase cancer risk also in non-metabolic patients. Indeed, regardless of adiposity, a high cancer risk can be determined by insulin resistance [57], dyslipidemia [107], and predicted basal metabolic rate [108], being indicative of the whole-body energy metabolism. Therefore, characterizing the obese state by systemic and tissue-specific measures could provide a more reliable identification of high-risk populations and represents a fascinating approach for the development of preventive and therapeutic strategies [109]. Additionally, the right anthropometric assessment may be crucial to better identify subjects at high risk of developing cancer among apparently healthy individuals just before they turn into patients. Strategies aimed at counteracting obesity epidemic represent a way of primary cancer prevention. Moreover, they may be a viable complementary option to improve prognosis in addition to canonic and unavoidable pharmacological treatments. Overall, under the assumption that the amount of WAT increases the risk of cancer and affects prognosis and mortality rates, reducing fat mass and enhancing energy expenditure may represent a fundamental non-pharmacological way to prevent cancer and improve survival.

### 3.1. Anthropometric Assessment: How to Identify Visceral Obesity

Since visceral fat is crucial in the obesity-cancer association, an ideal assessment of adiposity would consider both the amount and the site of deposition of adipose tissue. For instance, people who carry most of the fatty tissue in the abdominal region is 70% more prone to develop pancreatic cancer compared to those who bear it around the hips [110].

Computed tomography (CT) and magnetic resonance imaging (MRI) can accurately distinguish between SAT and VAT, assessing adiposity with a single image slice at pelvic level [111]. Unfortunately, they are expensive and complex instruments to be used routinely in clinical practice and cannot be easily applied to large datasets because of a time requirement and human resources [112]. To overcome these limitations, deep learning systems have been developing to reach a fully automated segmentation method [113], but the fascinating field of innovative work being done in this area, with technology advancing quickly to improve body compartment estimates, is beyond the aim of this review.

On the other hand, using BMI as a metric of adiposity in adults may lead to some methodological bias. Firstly, BMI is only a ratio of weight to height and does not distinguish fat mass from lean body mass or among SAT and VAT, nor apple- from pear-shaped body. Secondly, the timing and method of BMI assessment seem to be critical [114,115]. When BMI assessment is not anthropometrically measured, it is usually self-reported by patients who tend to tell lower weight and higher height [116]. Moreover, lower BMI values should not be considered healthy when due to unintentional weight loss during cancer development, cachexia, and undernutrition resulting from chemotherapy-induced nausea and vomiting [114]. Indeed, in some circumstances weight loss may be a predictor of poor survival as for colon cancer [115]. Other anthropometric measures may instead better reflect adiposity and could be useful in clinical practice to individuate patients with higher risk of cancer. For instance, Waist Circumference (WC) is measured horizontally midway in the distance of the superior iliac crest and the lower margin of the last rib, so far it better reflects deep visceral adiposity [117]. Indeed, according to International Diabetes Federation definition, essential criteria to diagnose Metabolic Syndrome is adiposity assessed by WC [31]. Furthermore, WC is a sensitive predictor for the risk of obesity-related cancer [6], since it has been associated with increased incidence in endometrial [106] and colon [118] cancers, without the gender difference highlighted when adiposity was assessed by BMI [119]. In the future, it may be intriguing to assess the risk of cancer in patients with increased WC who are not classified as obese according to their BMI [120]. A study on 1,564,218 participants showed that high WC and high Waist-hip ratio (WHR) correlate with increased pancreatic ductal adenocarcinoma mortality, independently from BMI [121]. WHR is the ratio between WC and hip circumference. Adding WHR to a multivariable model increases the diagnostic accuracy for detecting prostate cancer [122]. Moreover, WHR has been associated with the worst subtypes of breast cancer [123]. Also, high WHR and WC, rather than general obesity measured by BMI, were associated with an increased risk of developing pancreatic cancer [124] in a large prospective study. This different accuracy in detecting cancer risk according to anthropometric assessment is additionally highlighted by evidence about colorectal cancer risk: while the association of BMI is stronger in men than in women, WHR raises the risk in both sexes [118]. Lastly, a recent prospective study concluded that body shape, assessed by WC, WHR, and A Body Shape Index (ABSI), is positively associated with lung cancer risk [125], reverting the aforementioned obesity paradox according to which higher BMI may be protective. Additionally, adult weight variations have been studied as a better metric. Adult weight gain is a dynamic measure and reflects better than BMI continuous and cumulative influence on carcinogenesis that excessive adiposity may exert. A meta-analysis by Keum et al. [126] concluded that each 5 kg increase in weight is associated with an approximately 11% increased risk of breast cancer, 13% for ovarian cancer, and 39% for endometrial cancer, among non-HRT users post-menopausal women, and increases the risk of colon and kidney cancers as well.

### 3.2. Browning of Adipose Tissue: Remodeling Fat Amount

Brown adipocytes differ from white ones since they contain multilocular lipid droplets, more abundant cristae-rich mitochondria, and higher levels of uncoupling protein-1 (UCP-1), which reduces the proton gradient across inner mitochondrial membrane producing heat rather than producing ATP, as instead occurs in white adipocytes. In this manner, fatty acids and glucose are burned as substrates in BAT through a process known as adaptive non-shivering thermogenesis [127], which occurs under specific stimuli and significantly contributes to energy expenditure [128]. Initially identified only in newborns, recent studies have shown that a tissue with cold-induced activity is still present in adults [129] and its amount is inversely correlated with BMI [48], percentage of body fat and visceral fat [130]. In overweight or obese subjects this tissue is not only reduced, but is also less metabolically active [131]. It remains unclear if this reduced activity is a predisposing factor for obesity or an adaptative trait since weight loss in obese subjects has been associated with BAT recruitment [132]. Further studies have identified this cold-induced tissue be made of brite adipocytes [133]. As suggested by the name “brite”, literally brown-in-white, these cells share some characteristics of both brown and white adipocytes. Indeed, brite adipocytes are typical white adipocytes residing in WAT that, upon specific stimuli, switch their phenotype into brown-like adipocytes, in a process called browning [134]. After browning, brite adipocytes express thermogenic genes, specifically UCP-1, and increase their mitochondrial content, although they never reach the thermogenic capability of classical brown adipocytes [135]. It has not yet been fully elucidated if brite adipocytes derive from *de novo* differentiation of precursor cells or from trans-differentiation of mature white adipocytes [136], but probably both mechanisms may contribute to brite fat biogenesis [137], since roughly 40% of pre-adipocytes isolated in SAT has brite cells features [138]. Brown adipocytes consume glucose [131] and uptake FFAs from circulating lipoproteins [139]. Thus, increasing the amount of brite cells and upregulating pre-existing BAT not just increase energy expenditure [140], but may also improve glucose tolerance [141] and dyslipidemia, finally contributing to adiposity control [130]. Notably, also adipokines are involved in BAT metabolism. Leptin increases UCP-1 expression, and therefore may activate thermogenesis and stimulate fat oxidation [142]. Moreover, acting together with insulin on hypothalamic POMC neurons, leptin promotes WAT browning [143]. Conversely, adiponectin [144] and TNF-α [145] reduce BAT activity.

It is important to note that the recruitment of brite adipocytes from WAT reduces the net number of white adipocytes and, in conclusion, the fat amount. Indeed, mice with increased brite fat mass gain significantly less body weight and adiposity when fed with a high fat diet [146]. Since just visceral adiposity has been related to more metabolically active BAT [130] and given that is possible to activate BAT and pre-brite cells in obese people [131], pharmacological and non-pharmacological methods to stimulate browning [147] have been studied as possible strategies to reduce fat amount and potentially cancer risk. To achieve this goal, acting on physiological stimuli of browning may represent a powerful approach. For instance, stimulating adaptive thermogenesis in response to high calorie or high fat diets [148] or cold exposure might be a way to prevent or treat obesity [149]. As adrenergic stimulation by epinephrine and norepinephrine [150] have been detected among cold-induced pathways, also beta-3 adrenergic agonists, as mirabegron, can potentially enhance browning [151]. Furthermore, physical exercise, through irisin production, has been proposed as a possible way to increase BAT thermogenesis and browning [152]. Particularly, irisin induction as a consequence of PPAR-α activation likely represent the way by which fenofibrate induces browning in subcutaneous WAT [153]. Also PPAR- γ induction by rosiglitazone is potentially involved in browning [154], while liraglutide leads to body weight loss by activating BAT thermogenesis and WAT browning, acting on hypothalamic GLP-1 receptors, independently from food intake [155]. Among nutrients, fish oil intake has been proposed to increase thermogenesis-associated genes expression [156], while beige remodeling of SAT may be one of factors conferring beneficial anti-obesity effects to resveratrol [157]. Lastly, the discovery that depletion [158] or transplantation of every-other-day fasting treated mice microbiota [159] enhances browning of WAT, thus shedding a light on a new potential role of diet in shaping microbiota to reduce adiposity.

### 3.3. Lifestyle Interventions—Let Food Be Your Medicine

Since it is undoubtedly clear that obesity is one modifiable risk factor for tumorigenesis, lifestyle interventions addressed to reduce adiposity, including dietary changes and physical activity, play a pivotal role in cancer prevention [160]. For instance, pancreatic cancer risk was inversely associated with a healthy lifestyle assessed considering diet quality, physical activity, smoking status, alcohol consumption and anthropometry [161].

Intensive lifestyle intervention (diet modification and physical activity) also improves glycemic control and leads to weight loss [162] and to a significative reduction of obesity-related cancer risk [163].

Physical activity reduces body fat [164], and regular moderate exercise, even with no dietary intervention, reduced preferentially visceral fat in nonobese healthy women [165]. Additionally endurance training induced fat loss, particularly VAT, in obese women with MS, also reducing BMI and WC [166,167]. These findings are consistent with a meta-analysis by Vissers et al. [168] and with the results of a study in rats fed with a high-fat diet in which the beneficial effects of exercise training were more pronounced on VAT than on SAT [169]. Furthermore, physical activity decreases appetite, particularly in obese individuals, and ameliorates lipid profile since it increases HDL cholesterol and reduces TAG blood levels [170].

Consequently, thanks to its role in reducing VAT, physical activity should be recommended not only in preventing cancer, but also as a complementary approach to reduce adverse events and ameliorate pharmacological strategies in survivors [171].

Of note, not only weight loss *per se*, but also the kind, the amount and the timing of meals influence those pathogenetic mechanisms that are at the basis of the close connection between cancer and obesity. The European Prospective Investigation into Cancer and Nutrition (EPIC) study, a multicentre prospective cohort study with the aim of investigating the relationship between nutrition and cancer, has assumed a paramount importance in this research area. The World Cancer Research Fund/American Institute for Cancer Research (WCRF/AICR) [164] strongly recommends being a healthy weight, physically active, eat a diet rich in wholegrains, vegetables, fruit, and beans, limiting consumption of red and processed meat, sugar sweetened drinks, and alcohol. Although these recommendations are addressed to single individuals, policymakers should encourage and sensibilize about healthy behaviors and also provide economic means to give everyone access to healthy food, in consideration of the relative low cost of unhealthy foods. [172].

Although some foods (e.g., allium and broccoli) and micronutrients (e.g., selenium, vitamin D, carotenoids) have been studied for their anti-cancer properties [173], it has emerged that studying dietary patterns as a whole is more beneficial than considering single nutrients. Among various dietary patterns, the traditional Mediterranean diet (MD) seems to produce substantial health benefits [174]. The core of this diet is mainly vegetarian, lower in meat and dairy products, with moderate alcohol consumption, mainly in the form of wine. However, MD is not only a combination of foods, but a regular lifestyle and a traditional way of interacting with environment.

Analyzing MD composition led to consider the importance of fat total intake. It has been demonstrated that a low-fat dietary intervention significantly reduces cancer incidence, as seen in pancreas [175], whereas high fat diet (60% of total energy intake derived from fat) may increase cancer risk [176]. Paradoxically, MD displays a high fat content (30–40% of total energy intake), but with a higher percentage of unsaturated than saturated FAs, given that extra-virgin olive oil is the major source of lipid in this diet [177]. The ratio between unsaturated and saturated FAs is important to determine the effect of this lipid source. On molecular level, high content of saturated FAs is more prone to create an inflammatory environment and perpetrate damage to cell membranes. This is consistent with the findings that extra-virgin olive oil exerts beneficial effects on chronic inflammatory disorders that may eventually lead to cancer [178]. Moreover, a high-unsaturated FAs diet improves adiponectin levels [179], thus limiting the detrimental process. Furthermore, it has been observed that replacing lard (rich in saturated FAs) with soybean oil (high content of unsaturated FAs) in high-fat diet alleviates obesity-related inflammation and insulin resistance by reducing macrophage infiltration into AT [180].

As discussed before, chronic inflammation, insulin resistance, and dysregulation in adiponectin levels characterize obesity, and specifically are associated with VAT. By eliciting the reshape of VAT, MD may prevent these metabolic alterations, therefore limiting the metabolic disorders and associated diseases.

Indeed, MD has been associated with a significant reduction in central obesity [181] and people with greater adherence to MD showed significantly lower WC [182]. Of note, this inverse association has been attributed to VAT and not to SAT [183]. Furthermore, some RCTs studied the MD effects for 3 months [184], 1-year [185], and 2-years [186] interventions in obese and overweight patients showing reduction in body weight, BMI, WC, and body fat. These effects are probably more pronounced in long-term interventions. Indeed, a Spanish RCT showed that a long-term high-vegetable-fat MD, even with an unrestricted-calorie, was associated with less gain in central adiposity compared with a control diet. [187]. Although further studies are needed to clarify if the effects of a MD pattern on obesity are specifically due to reduction of visceral fat, since VAT is highly associated with most of the metabolic effects of obesity [188] and obesity represents a risk factor for cancer, we speculate that the MD anticancer role may be related to its beneficial effects on visceral adiposity.

Several evidences highlight that MD reduces overall risk of cancer, in a dose-response manner [189]. More specifically, MD has been associated with reduced incidence of colorectal [190], gastric [191], high aggressive prostate [192] and breast cancer [193], and particularly receptor negative breast cancer in post-menopausal women [194]. Moreover, MD has been favorably associated with reduced cancer mortality [195]. In this contest, there are two hurdles to overcome. Firstly, these evidences are mainly based on many prospective and fewer interventional studies. Secondly, there is no unique index to assess adherence to MD regimen. Furthermore, it rests unclear which component of the MD mostly contributes to its beneficial effects, although the benefits of MD are mainly driven by higher intake of fruits, vegetables, and whole grain [196]. For instance, olive oil polyphenols, red wine resveratrol, and tomato lycopene are able to reduce colorectal cancer initiation and progression [197]. Moreover, high dietary fiber intake, as consequent of whole-cereals meals, a cornerstone of MD, is inversely associated with colorectal [198], liver and stomach cancer [199].

One of the proposed mechanisms by which MD displays a protective role against cancer relies on its antioxidant effects, which mainly reduce DNA and molecular damages that are implicated in tumorigenesis [200]. For instance, the antioxidant phenolic compounds present in olive oil are powerful inhibitors of free radical generation [201]. Moreover, MD displays anti-inflammatory and anti-aggregating MD properties [202], thus contraposing to Western-type diet, rich in red meat, high saturated-fat dairy products and refined grains, which has been related to increased inflammatory markers [84]. In the future, the identification of the metabolites influenced by a given nutritional intervention and the characterization of the complex metabolic effects of nutrients or foods through metabolomics may be of substantial help to identify novel risk factor for tumor [203], as it has occurred for prostate cancer [204].

Dietary characteristics of MD may also explain its protective role described in diabetes [205]. Indeed, a typical MD composition improves insulin sensitivity in patients without preexisting diabetes and this effect is probably linked to the increased amount of unsaturated FAs intake [179]. Therefore, given that MS, hyperinsulinemia, and obesity are risk factors for tumor initiation and progression, and since the MD-style pattern reduces the development of MS and central obesity by acting on lipids levels and glucose metabolism, it is reliable argument that MD may be protective against cancer onset and growth [182,206].

Lastly, MD features are associated with positive modulation in microbiome [207]. Gut microbiota has nowadays been identified as a true organ, hidden in the host, which dynamically responds to environmental factors, among which the diet plays an essential role. Dietary contents modulates microbiome composition [208], which in turn influences nutrients absorption, impairs host energy homeostasis regulating lipid and glucose metabolism, and provides immunomodulatory effects, through a crosstalk with the host [209,210]. Dysbiosis of the commensal microbiota is implicated in the pathogenesis of several diseases, including obesity and cancer, probably by chronic inflammation [211]. A possible explanation for this low-grade chronic inflammation also comes from animal models of diet-induced obesity in which dysbiosis increased gut permeability letting a low grade metabolic endotoxemia [212]. Notably, dysbiosis has been associated with colorectal cancer and with tumors arising in organs distant from the gut. Therefore, it is easy to infer that by modifying microbiome, MD intervention would be beneficial to counteract cancer progression.

Altogether, it appears quite clear the key role played by dietary interventions, not only in reducing adiposity *per se*, but also in reverting the inflammation and metabolic impairment that connects obesity to cancer. Nonetheless, in addition to the source and the properties of some food groups, and to their combination in dietary-pattern, the timing of meals and the length of fasting and feeding periods have also been studied as modulators of metabolic pathways and oxidative-stress. These hypotheses take origins from the findings that calorie restriction (CR), a reduction in energy daily intake with maintained number and frequency of meals, delays age-related pathologies and increases lifespan. Intriguingly, the implementation of a CR approach in mice has been shown to stimulate browning in VAT, resulting in loss of weight and visceral adiposity mainly due to a decrease of adipocytes size, which dampens the inflammatory processes overall [213,214]. By contrast, a CR approach for 8 weeks in obese individuals results in a negative regulation of browning in SAT [215].

In general, the weight decrease observed in overweight subjects after a CR regimen is associated with a great extent to VAT reduction rather than the SAT one [216,217]. This correlated also with a decrease of some biochemical parameters, including total cholesterol, triglycerides, and fasting glycaemia [216]. In line with this, two different studies on obese individuals underwent CR diet for 14 days demonstrated a significant reduction of the VAT, but not the SAT, probably due to a dissimilar responsiveness and physiological characteristic of the adipose cells in the two compartments: differently from the SAT, VAT cells display induction of lipid metabolism related genes in response to fasting [218,219]. Interestingly, when the effect of CR on body fat distribution was investigated independently or in combination with regular exercise, it emerged that the body weight reduction and the VAT loss were not statistically different between the different groups [220,221], therefore highlighting the cruciality of CR in the reduction of visceral adiposity.

By boosting the regenerative capacity of stem cells as seen in multiple rodent tissues [222], decreasing metabolic rate and oxidative damage [223], CR plays also a protective role in tumorigenesis [224]. In rat models of colon cancer, CR decreased leptin level and concomitantly reduced tumor growth [83]. Unfortunately, a long-term CR regimen is difficultly feasible in clinical practice, because of adverse events. Consequently, other strategies have been studied to achieve the same goals with a better profile in patient safety and adherence. In this contest, starvation has been shown to promote stress resistance and longevity in mice and humans [225]. One general mechanism of action of fasting is that it triggers adaptative cellular stress response, which results in a major efficiency in counteracting diseases; in addition, among the major effects of fasting relevant to aging and disease there is decrease in IGF-1, IGFBP-1, glucose, and insulin levels. Different methods of fasting (Table 2) have been pinpointed to decrease weight, delay aging, reduce tumorigenesis, and protect mice from chemotherapy drugs when fasting cycles were associated with treatments [226].

Moreover, in humans, fasting seems to reduce common chemotherapy associated side effects [227]. Intermitting fasting (IF) usually refers to a water-only or very low-calorie period lasting less than 24 h, followed by a normal feeding period of 1 or 2 days. In animal models, IF ameliorates lifespan and mitigates a wide range of chronic diseases, including obesity, insulin resistance, diabetes, and cancer, through inducing a metabolic switching and improving cellular stress resistance [228]. Every other day feeding (EODF) [229] is a kind of IF program that extends lifespan, stimulates browning, and reduces obesity through its interactions with gut microbiota, as mentioned before [159]. Time-restricted feeding (TRF) requires restricting the timing of meals, without regard to their caloric content, to a time window of few hours. Studies on TRF have also highlighted the importance of respecting circadian rhythm to maintain optimal metabolic function [230]. Proposing fasting program instead of CR is potentially more fruitful in clinical translation to prevent cancer and ameliorate therapy responsiveness and tolerance, since starvation seems to be more powerful in determining metabolic changes, such as IGF-1 and glucose dramatic decrease [231], and may be more suitable to be followed by patients [225].

Dietary regimens that provide a normal or high caloric content but are able to induce typical fasting metabolic pathways have been proposed, i.e., fasting mimicking diets (FMDs) [232]. Among these FMDs, ketogenic diet (KD) has been associated with weight loss and has been proposed to protect against cancer and as a therapeutic anti-cancer agent [233,234]. KD is a low carbohydrate (usually less than 50 g/day), high fat, and protein regimen that lowers insulin levels and induces ketone bodies over-production in the liver, mimicking the metabolic state of fasting. The acute metabolic benefits of KD principally relies on the metabolic switch from glucose metabolism towards fatty acid oxidation. High protein diets are considered a powerful strategy to improve body weight management and decrease fat mass in both normal and obese individuals [235,236]. Very-low calorie KD (VLCKD) have been generally associated with body weight loss, reduction of visceral adiposity, and improvement of lipid profile, as well as cardiac parameters [237,238,239]. Moreover, VLCKD induces a greater reduction in body weight and WC than a standard low-calories diet, mainly through a selective re-shaping of VAT. Intriguingly, these modifications are still effective after 2 years from diet intervention [240]. Similar results have been observed also in women with endometrial or ovarian cancer that underwent KD or a diet high in fiber and low in fat: change in visceral fat depots was grater in the KD group with retention of the lean mass and decrease of serum fasting insulin [241]. Of note, the remodeling of VAT caused by short time KD could be related to changes in the mix of innate immune cells in this fat depots, which result in a reduced inflammation and in metabolic health improvement [242]. However, whereas the short-term KD approach is beneficial, and does not display cytotoxic effect or increase oxidative stress, long-term continuous KD induces obesity and glucose intolerance, as well as inflammation in visceral fat stores, thus pointing at the need of a managed weighed control during this dietary regimen [237,242].

Brandhorst et al. have proposed a FMD with low protein, low sugar, and relatively high fat content, coupled with a periodic fasting (PF) program. PF is another form of starvation that lasts 2 or more days and is separated from the next cycle by at least 1 week of normal feeding, with well-established beneficial effects on inflammation. So far, this kind of FMD lasts 4 days and provides 10–50% of the normal caloric, showing potent effect on lifespan and health span, leading rats to weight loss by reducing visceral fat amount, reducing inflamed tissues, and decreasing neoplasms development. In a pilot trial on humans, similar FMD reduced fasting blood glucose, circulating IGF-1 and IGFBP-1 levels, and led to significant fat loss, without affecting lean body mass [243]. Therefore, PF and FMD have an exceptional potential also in enhancing disease treatment in patients at risk for cachexia. However, although the recent developments in this field have generated a lot of excitement, some doubts exist with regard to its translation in clinical practice, as more robust clinical experiments are needed to support findings in animal models. From a practical perspective, a first hurdle to overcome is the feasibility of such a revolutionary diet intervention in a western routine made of always available food, nighttime eating and hypercaloric snacks. Moreover, some fasting methods are unfeasible in the long run and many side effects could be reported if longer studies were performed in humans. Furthermore, each of us has a peculiar metabolic baseline, and dietary intervention should be individualized as much as possible. For instance, fasting should not be proposed to those with deficiencies in metabolic pathways or in those patients that require a nutritional support not consistent with the composition of a FMD. Furthermore, personal preferences and metabolic considerations might inform individualized tailoring of dietary interventions [186] to be more easily proposed to patients and ameliorate their compliance.

## 4. Conclusions and Recommendations

In the future, it will be unavoidable to summarize evidence from epidemiological data and experiments in animal models, with clinical trials and a deep knowledge in dysregulated metabolic pathways, in order to provide targeted interventions in lifestyle, nutrients, and drugs, able to prevent and reduce adiposity and to break down its link with cancer.

Dietary interventions provide an economically viable, non-pharmacological approach for eliciting beneficial adaptation in body composition, decreasing VAT and improving weight loss. A healthy lifestyle based on the combination of appropriate diet approach and physical activity represents a preferential way to dampen the negative sequalae of inflammation, and halt cancer onset and progression.

Findings about dietary extensive influence on metabolism and cancer let us speculate whether different habits in dietary regimens affect the effectiveness and tolerance of some drugs traditionally used to treat cancer. In keeping with this, although projecting drugs which elegantly affect a single molecule or a specific pathway may be intriguing, an integrated therapeutical approach is required.

## Figures and Tables

**Table 1:** Summary of evidences for increased cancer risk in obesity.

Cancer Site or Type	Renehan et al. ^1^ (2008)	IARC Working Group ^2^ (2006)	Wang et al. ^3^ (2016)
Thyroid	Men (*p* = 0.02)	Yes	Men (*p* < 0.0001)
	Women (*p* = 0.001)		Women (*p* = 0.728)
Kidney	Men (*p* < 0.0001)	Yes	Men (*p* < 0.0001)
	Women (*p* < 0.0001)		Women (*p* < 0.0001)
Colon	Men (*p* < 0.0001)	Yes	Men (*p* < 0.0001)
	Women (*p* < 0.0001)	(colorectal)	Women (*p* = 0.005)
Rectum	Men (*p* < 0.0001)		(colorectal)
Esophagus	Men (*p* < 0.0001)	Yes	Men (*p* < 0.0001)
	Women (*p* < 0.0001)	(adenocarcinoma)	Women (*p* = 0.041)
	(adenocarcinoma)		(esophagus and stomach)
Stomach	-	Yes	
Multiple Myeloma	Men (*p* < 0.001)	Yes	-
	Women (*p* < 0.0001)		
Leukemia	Men (*p* < 0.0001)	-	-
	Women (*p* = 0.01)		
Non Hodgkin Lymphoma	Men (*p* < 0.0001)	-	-
	Women (*p* = 0.01)		
Melanoma	Men (*p* = 0.04)	-	-
Gallbladder	Women (*p* = 0.04)	Yes	-
Pancreas	Women (*p* = 0.01)	Yes	Men (*p* < 0.0001)
			Women (*p* = 0.014)
Liver	-	Yes	Men (*p* < 0.0001)
			Women (*p* = 0.9)
Meningioma	-	Yes	-
Ovary	-	Yes	Women (*p* = 0.009)
Prostate	-	-	Men (*p* < 0.0001)
Endometrium	Women (*p* < 0.001)	Yes	-
		(corpus uteri)	
Postmenopausal Breast cancer	Women (*p* < 0.0001)	Yes	Women (*p* < 0.0001)

^1^ Increased RR per 5 kg/m^2^ increase; ^2^ Increased Relative Risk of the highest BMI category evaluated vs Normal BMI (95% Confidence Interval), no gender difference; ^3^ Increased RR per 5 kg/m^2^ increase.

**Table 2:** Proposed methods for fasting.

Method	Description
Every-Other-Day Fasting	Food is withdrawn for 24 h on alternate days, with water provided ad libitum. Overall calorie intake need not be limited.
Time-Restricted Feeding	It restricts the timing of meals, without regard to their caloric content, to a time window of few hours in a day.
Periodic Fasting	It lasts 2 or more days and is separated from the next cycle by at least 1 week of normal feeding.
Brandhorst	It lasts 4 days and provides 10–50% of the normal caloric intake
Fasting Mimicking Diet	Periodic cycle of diets that provides a relatively high caloric content but mimics effects of fasting.
